# Identification of sex determination locus in sea cucumber *Apostichopus japonicus* using genome-wide association study

**DOI:** 10.1186/s12864-022-08632-3

**Published:** 2022-05-23

**Authors:** Yixin Wang, Yujia Yang, Yulong Li, Muyan Chen

**Affiliations:** 1 The Key Laboratory of Mariculture, Ministry of Education, Ocean University of China, Qingdao, China; 2 Key Laboratory of Marine Ecology and Environmental Sciences, Institute of Oceanology, Chinese Academy of Sciences (CAS), Chinese Academy of Sciences (CAS), Qingdao, China

**Keywords:** Sex determination, Echinoderm, *Apostichopus japonicus*, GWAS, Sex-specific SNPs

## Abstract

**Background:**

Sex determination mechanisms are complicated and diverse across taxonomic categories. Sea cucumber *Apostichopus japonicus* is a benthic echinoderm, which is the closest group of invertebrates to chordate, and important economic and ecologically aquaculture species in China. *A. japonicus* is dioecious, and no phenotypic differences between males and females can be detected before sexual maturation. Identification of sex determination locus will broaden knowledge about sex-determination mechanism in echinoderms, which allows for the identification of sex-linked markers and increases the efficiency of sea cucumber breeding industry.

**Results:**

Here, we integrated assembly of a novel chromosome-level genome and resequencing of female and male populations to investigate the sex determination mechanisms of *A. japonicus*. We built a chromosome-level genome assembly AJH1.0 using Hi-C technology. The assembly AJH1.0 consists of 23 chromosomes ranging from 22.4 to 60.4 Mb. To identify the sex-determination locus of *A. japonicus*, we conducted genome-wide association study (GWAS) and analyses of distribution characteristics of sex-specific SNPs and fixation index F_ST_. The GWAS analysis showed that multiple sex-associated loci were located on several chromosomes, including chromosome 4 (24.8%), followed by chromosome 9 (10.7%), chromosome 17 (10.4%), and chromosome 18 (14.1%). Furthermore, analyzing the homozygous and heterozygous genotypes of plenty of sex-specific SNPs in females and males confirmed that *A. japonicus* might have a XX/XY sex determination system. As a physical region of 10 Mb on chromosome 4 included the highest number of sex-specific SNPs and higher F_ST_ values, this region was considered as the candidate sex determination region (SDR) in *A. japonicus*.

**Conclusions:**

In the present study, we integrated genome-wide association study and analyses of sex-specific variations to investigate sex determination mechanisms. This will bring novel insights into gene regulation during primitive gonadogenesis and differentiation and identification of master sex determination gene in sea cucumber. In the sea cucumber industry, investigation of molecular mechanisms of sex determination will be helpful for artificial fertilization and precise breeding.

**Supplementary Information:**

The online version contains supplementary material available at 10.1186/s12864-022-08632-3.

## Background

Sexual determination and differentiation is one of the fascinating areas of study in developmental biology, evolutionary biology and ecology, known as "the queen of evolutionary biology" [1]. In many Eukaryotic lineages, the mechanisms underlying sex determination are surprisingly complicated and diverse across taxa, closely associated with the independent evolutionary process of sex chromosomes [2, 3]. Sex chromosome evolution includes heteromorphism of sex chromosomes and faster turnover of sex chromosomes [4, 5]. The heteromorphic sex chromosomes evolve from a pair of autosomes due to recombination suppression [6]. However, in amphibians, reptilians, and fishes, highly diverse sex determination systems exist due to the frequent location transition of sex-determinating genes and high turnover rates of sex chromosomes [7, 8]. Homomorphic sex chromosomes are cytologically indistinguishable and equally crucial to the sex-determination system, which might be of the same evolutionary age as heteromorphic sex chromosomes [9]. Many taxonomic groups with homomorphic sex chromosomes possess sex-specific regions or sex-specific expressed genes to determine the genetic sex [10]. Therefore, investigating homomorphic chromosome systems requires high-quality genomic resources and optimized bioinformatics analysis tools to identify potential sex determination factors and gain insights into the evolution of sex chromosomes.

Sex determination mechanisms consist of two main types in terms of major sex determination factors, genetic sex determination (GSD) and environmental sex determination (ESD) [11, 12]. Sex can be determined by a transition between GSD and ESD, where GSD and ESD are more like two ends of a continuum [13, 14]. In mammals (except some rodents) and birds, their sex determination mechanism is dominated by GSD, which has highly differentiated sex chromosomes and shared master sex determination genes [15]. In the ESD system, temperature-dependent sex determination (TSD) is one of the most common types, which has been discovered in some reptiles, becoming a specific feature of this group among tetrapods [9, 16]. Interestingly, teleost fishes have the most diverse sex determination mechanisms among vertebrate species, including GSD (e.g., ♀XX/♂XY, ♂ZZ/♀ZW, ♀XX/♂X0, ♀X1X1X2X2/♂X1X2Y, ♀XX/♂XY1Y2, etc.) and ESD (e.g., temperature, pH, behavior, population density, oxygen concentration, and social status, etc.) [2, 17, 18]. Invertebrates also have diverse reproductive modes and sex determination systems [19]. For instance, in Hymenoptera such as ants and bees, the homozygous or heterozygous sex determination sites induce males or females [4]. And in *Caenorhabditis elegans* and *Drosophila melanogaster*, sex is controlled by the ratio of X chromosomes to autosomes (X: A ratio) [20]. Notably, an interesting pattern shared by known sex determination mechanisms among different taxa is that the master sex determination genes were predicted diverse, but the downstream genetic networks associated with sex determination or differentiation were found being conserved [12, 21, 22].

Echinoderms are one of the closest groups of invertebrates to chordate, with particular evolutionary classification and phylogeny [23]. Reproduction modes are diverse in echinoderm species, including asexual multiplication (budding, fragmentation, fission), parthenogenesis, hermaphroditism (simultaneous hermaphroditism, protandry-first male and then female), and dioecy [19]. Therefore, echinoderms are excellent research objects for studying sex determination and sexual plasticity. However, several features of echinoderm genome render sequence assembly difficult, including large genome size, a large number of low frequency repeats, high heterozygosity, and high genome sequence variation [24, 25]. Besides, the knowledge of sex determination in echinoderms is still very limited, mainly focusing on karyotype analysis [26–28], sex-associated markers [29, 30], linkage-mapping analysis [31–33], and gonadal transcriptome [34–37].

Sea cucumber *Apostichopus japonicus* is a benthic echinoderm and important economic and ecologically aquaculture species in China. Like most sea cucumbers, *A. japonicus* is dioecious, and no phenotypic differences between males and females can be detected before sexual maturation. Recently, using 2b-RAD sequencing, several sex-specific tags were found for identifying the genetic sex of *A. japonicus* [29]. Interestingly, the sex-associated SNP markers showed female homogamety and male heterogamety, which indicates *A. japonicus* has an XX/XY sex-determination system [29]. In addition, recent studies of male and female *A. japonicus* using transcriptomic, proteomic, and metabolomic techniques have provided technical support for studying sex determination and differentiation, and gonadal development [36–38]. Up to now, a chromosome level reference genome of *A. japonicus* was still absent, which is important to explore the sex determination mechanism of *A. japonicus* without heteromorphic chromosomes. However, Hi-C technology has made it possible to assemble the reference genome at the chromosome level.

In this study, we assembled a chromosome-level genome of *A. japonicus* by Hi-C technology and continued with female and male population resequencing to study the genetic mechanisms of sex determination of *A. japonicus*. We conducted a genome-wide association study to search for sex-associated regions of *A. japonicus*. Using principal component analysis (PCA), chromosome 4 was observed closely associated with sex determination. Regarding the distribution characteristics of sex-specific SNPs and the selection signatures of Fixation index F_ST_, our results further indicated that *A. japonicus* might have an XX/XY sex-determination system, and 15–25 Mb region on chromosome 4 might be the candidate sex-determination region (SDR). This study will help reveal the genetic mechanisms of sex determination in sea cucumber and provide insights into a deeper understanding of the sex determination mechanisms in echinoderms. The knowledge we gain from this study will be useful for artificial fertilization in the breeding industry.

## Materials and Methods

### Hi-C library construction and chromosome level genome assembly

The current scaffold-level *A. japonicus* genome (assembly version: ASM275485v1) was sequenced from a male individual [39]. In the present study, we used the gonad of a male sea cucumber to construct Hi-C sequencing library. Briefly, one gram of gonad sample was immersed in 35 ml ice-cold 1*PBS and 2 ml 36% formaldehyde with final concertation of 2% for crosslinking. The crosslink reaction was incubated for 30 min at room temperature. Next, the fixed tissue was then washed with ice-cold 1*PBS, homogenized with tissue lysis buffer, and digested with the restriction enzyme Hind III. The isolated DNA was repaired with biotinylated residues and blunt-end ligated together in situ using T4 DNA ligase. After that, DNA was sheared into 300-700 bp fragments by Covaris S220. The dATP was then attached to the 3’end of DNA fragments, and DNA was retrieved and quantified by Qubit 3.0. The Illumina pair-end adapters were ligated to the DNA fragments by T4 DNA ligase. Further, Hi-C library was constructed by PCR and quality controlled by Qubit 3.0, LabChip GX Touch, and Q-PCR before sequencing. The sequencing of Hi-C library was conducted on Illumina HiSeq sequencing platform. After data filtering by HiC-Pro v3.0.0 [40], approximately 85.03 Gb clean data were generated from Hi-C library sequencing. After comparing with the scaffold-level genome by Juicer v1.6 [41], 3D-DNA (https://github.com/aidenlab/3d-dna) was used to primarily correct misjoin in the scaffold [42]. The results from 3D-DNA were preliminarily clustered, sequenced, and directed using ALL-HiC v0.9.8 [43, 44]. Juicebox was then used to adjust, reset, and cluster the genome sequence. Finally, the genome quality was evaluated by BUSCO v5.0.0 based on 954 genes of metazoa odb10.2021–02-17 [45].

### Gene prediction and annotation

To search for tandem repeats in the genome assembly, we used TETools v1.3.1 (https://github.com/Dfam-consortium/TETools), a Dfam TE Tools including RepeatMasker, RepeatModeler, and coseg, and a de novo repeat library was built and then was trained using transcriptome data of *A. japonicus* (BioProject: PRJNA723369; SRA: SRR17083776) as hints file (http://funannotate.readthedocs.io.). Subsequently, gene prediction and annotation were carried out using Funannotate pipelines version 1.8.5, including software for prediction, AUGUSTUS, GeneMark, gilmmerhmm, Snap, and tRNAscan-SE; for annotations: Pfam 34.0 (data:2021–03), Uniprot (data:2021–01), EggNog (emapper DB: 2.0), MEROPS Release 12.4, dbCAN HMMdb release 9.0 (data:2020–8-4), BUSCO (data: 2021–04-06), SignalP -5.0, InterProScan5 84.0 (data:2021–02-11).

### Whole-genome re-sequencing and SNP calling

A total of 60 sexually mature individuals (sex ratio 1:1) of *A. japonicus* were collected from the coast of Jiao Zhou Bay of the Yellow Sea, Qingdao, Shandong Province, China. The sex of each individual was determined by visual examination of the gonad after dissection. Genomic DNA was extracted from muscle samples of collected sea cucumber using DNeasy Blood & Tissue Kit (Qiagen, Valencia, CA, USA). DNA quality was identified by agarose gel electrophoresis. Whole genome sequencing libraries (insert size ~ 350 bp) were constructed following Illumina standard protocol. In brief, genomic DNA was randomly sheared, end repaired, and adaptor added. Fragments with both P1 and P2 adaptors (containing individual index) were enriched by high-fidelity PCR. The final libraries were sequenced on the Illumina HiSeq X Ten System (150 bp paired-end) with a mean coverage of ~ 10 × at Novogene, Co. Ltd., Beijing. Raw reads were preprocessed for quality control and filtered using fastp 0.18.0 [46]. High-quality paired-end reads were mapped to the reference genome using BWA-MEM2 [47]. The alignment outputs (BAM files) were sorted, and duplicated reads were removed using samtools v.1.12 [48]. Single nucleotide polymorphisms (SNPs) were called using GATK 4.0 (Genome Analysis ToolKit, Broad Institute, USA) and filtered by vcftools with the following parameters: “–maf 0.05 –min-meanDP 6 –max-meanDP 300 –minGQ 20 –max-missing 0.9 –min-alleles 2 –max-alleles 2 –remove-indels” [49].

### Whole‑genome sequence association analysis

After genotype-imputation by Beagle version 4.1 [50], whole genome association analysis with a specific focus on sex was conducted using plink v1.90b6.21 [51]. The output vcf files were obtained from SNP calling from female and male resequencing. The Manhattan plot was subsequently drawn using an R package named ‘CMplot’ [52]. In the present study, the significance threshold was set as − log10(*P*) = 6.0 to identify the significant sex-associated SNPs. To search for physical regions associated with sex in the genome, we summarized the chromosome-level statistics of sex-associated SNP density per unit chromosome length in the *P*-value threshold (*P* < 1-e6). Finally, the SnpEff 5.0e was used to predict the potential functional effects of SNPs [53]. SnpEff helps annotate SNPs in terms of effect impact, function class, location in gene structure. For instance, according to different functional class, these effects can be roughly divided into three types: missense effect, nonsense effect, and silent effect.

### Identification of candidate sex-determination region

To detect the sex-specific genetic variations and distinguish two sexes in sea cucumber, we performed principal component analysis using different sets of SNPs (including genome-wide SNPs and SNP sets on chromosome 4, 9, 17, and 18) by Genome-wide Complex Trait Analysis (GCTA version 1.93.2) [54]. Two additional methods were used to provide further evidence of candidate SDR: one was based on sex-specific SNPs, and the other one was based on F_ST_ values. The sex-specific SNPs were called by a Perl script (https://github.com/lyl8086/find_sex_loci) [55]. To qualify for a sex-specific SNP, we modified the parameters from Li et al., the SNP should be required with a minimum of 15 heterozygotes in one sex and no heterozygotes in another sex. The second method used in this study was calculating allele frequency differences between two sexes through estimating the Fixation index, F_ST_ using VCFtools software (version 0.1.16) [49]. The F_ST_ values exceeding 0.25 indicated very significant genetic differentiation [56].

### Transcriptome analysis of sex-biased genes within sex-determination region

The RNA-Seq datasets of sexually mature female and male gonads were downloaded from NCBI (NCBI Accession Number: SRP271890). The HISAT 2.2.1 package was used to map filtered reads against the chromosome-scale genome assembly AJH1.0. The alignment files were sorted and indexed using samtools v.1.12 [48]. The FPKM values were calculated using the featureCounts software to quantify changes in gene expression [57]. The differential expression analysis between females and males was performed by DESeq2 [58]. The differentially expressed genes (DEGs) were filtered with log2|FoldChange|> 1 and padj values < 0.05 (Benjamini and Hochberg method). Subsequently, the DEGs were subjected to enrichment analysis of Gene Ontology and KEGG pathways using the R package ‘clusterProfiler’ [59]. Finally, a heatmap of sex-biased DEGs within the candidate sex determination region was plotted using the R package ‘pheatmap’. Moreover, the genes located in the candidate SDR were annotated by local blast against the Uniprot and NCBI non-redundant databases.

## Results

### Chromosome-scale genome assembly and gene annotation

**Fig. 1:**
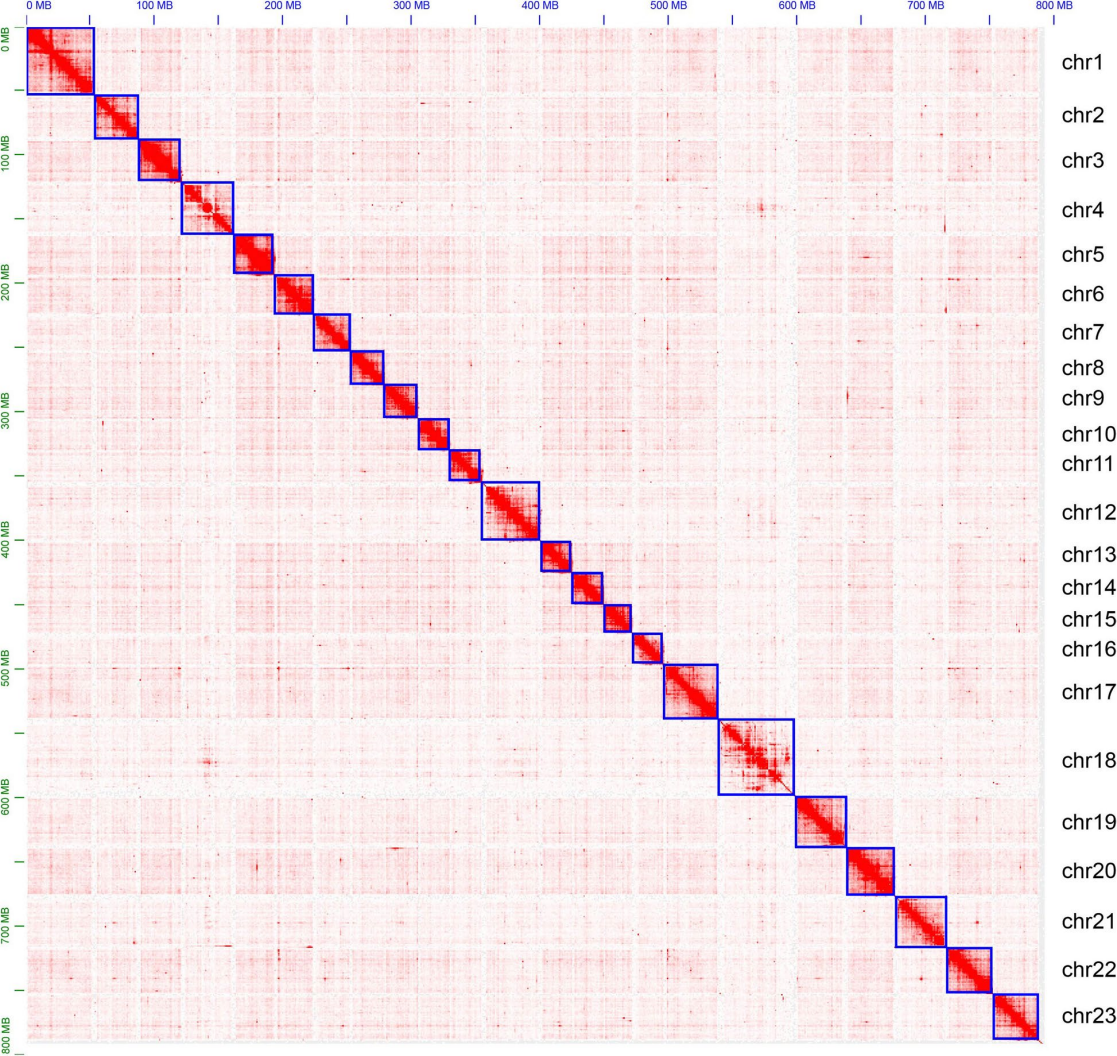
Contact map of Hi-C interactions among chromosomes in *A. japonicus* generated by Juicebox software

**Fig. 2:**
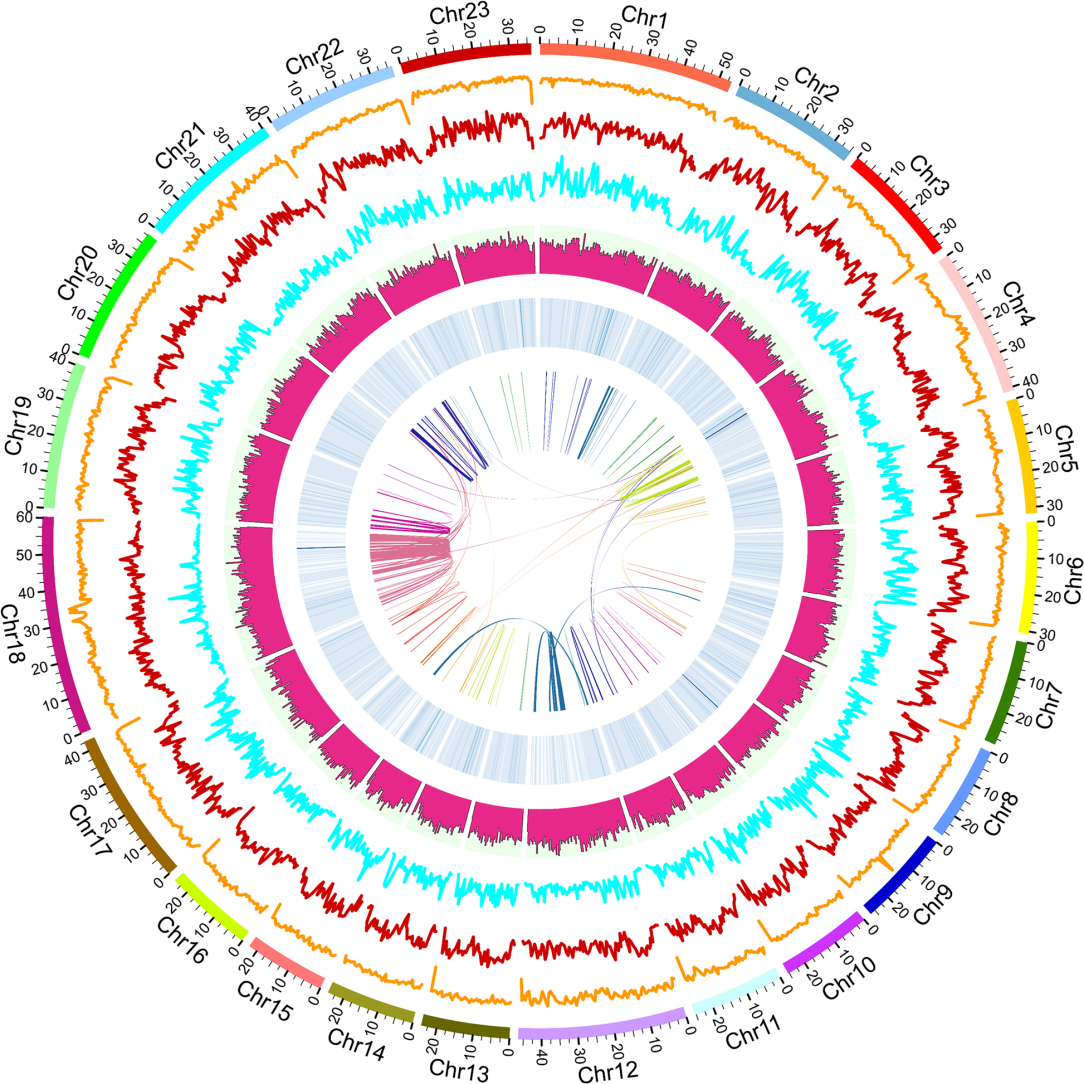
Circos plot of the global landscape of genomic features and genetic variations in AJH1.0 genome assembly. The tracks from outside to inside represent (**1**) 23 chromosomes of AJH1.0 genome assembly (Mb). (**2**) Distribution of GC content with a sliding window of 500 kb. (**3**) Distribution of INDEL density with a sliding window of 500 kb. (**4**) Distribution of SNP density with a sliding window of 500 kb. (**5**) Distribution of repeat elements with a sliding window of 500 kb. (**6**) Distribution of gene density with a sliding window of 500 kb. (**7**) Relationship between syntenic blocks in AJH1.0 genome assembly

The Hi-C library was sequencing by the Illumina HiSeq platform, and we obtained approximately 85.03 Gb clean data (~ 108 × genome coverage) with Q30 up to 91.33%. Our chromosome-level genome of *Apostichopus japonicus* AJH1.0 was assembled based on the previous scaffold-level genome (Zhang et al., 2017) using Hi-C technology. The genome assembly AJH1.0 contained 98.8% of the total sequences. As indicated in the Hi-C interaction heatmap (Fig. 1), AJH1.0 consisted of 23 chromosome-level scaffolds with lengths ranging from 22.4 to 60.4 Mb (Fig. 2 and Supplemental Table 1). BUSCO was then used to evaluate the integrity of the genome assembly, and the results indicated that 90.3% of the conserved genes were found in AJH1.0 (Supplemental Table 2).

The global landscape of genomic features and genetic variations in *A. japonicus* were presented in a circos plot, including GC content, INDEL density, SNP density, repeat elements density, gene density, and chromosomal collinearity (Fig. 2). After repeat masking, a total of 28,574 protein-coding genes were annotated using funannotate pipeline in *A. japonicus* genome, including 23,230 sequences (Pfam), 1,520 sequences (UniProt/EggNog), 1,097 sequences (MEROPS), 298 sequences (dbCAN (CAZymes) databases), 943 sequences (BUSCO) and 1,486 sequences (SignalP).

### Whole genome resequencing of both sexes

To investigate the sex determination system of *A. japonicus*, we obtained a total of 662.8 Gb raw data (~ 13.8 × genome coverage) from 60 sexually mature individuals (30 females and 30 males) (Supplemental Table 3). After quality filtering, approximately 638.47 Gb of clean reads (96.3% of raw reads) were aligned against the reference genome assembly AJH1.0, and the mapping rates per individual ranged from 96.5% to 97.4%. The average mapping rate was 97.08%.

A total of 73,222,019 SNPs were called from 60 individuals using GATK pipeline, and 3,926,865 SNPs were retained after quality filtering. We calculated genome-wide F_ST_ values of the above SNPs from female and male populations and observed no significant genetic structure or population subdivision between sexes at the whole-genome level. The mean F_ST_ and weighted F_ST_ between two sexes were 0.00049725 and 0.0009126, respectively.

### Genome-wide association studies and identification of sex-associated locus

**Fig. 3:**
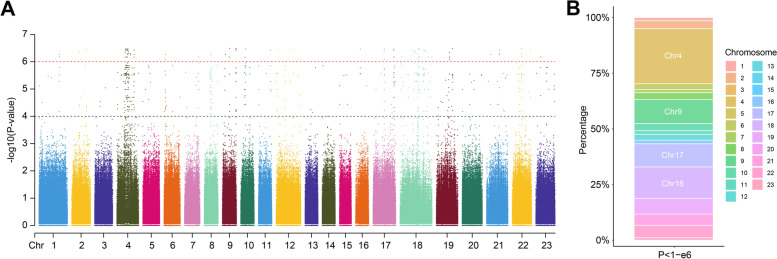
Genome-wide association analysis and chromosome distribution of sex-associated SNPs. (**A**) Manhattan plots depicting GWAS results associated with sex of *A. japonicus*. The x-axis shows the physical location of SNPs across the 23 chromosomes, and the y-axis shows the − log10 (*P*-value). The black threshold line represents *P*-value = 1-e4, the red threshold line represents *P*-value = 1-e6. (**B**) Chromosome distribution of sex-associated SNPs by GWAS. The x-axis depicts P-value thresholds (*P* < 1-e6), and the y-axis depicts the percentages of sex-associated SNPs per unit chromosome length for each chromosome

GWAS analysis was conducted to reveal the distribution of sex-associated locus in *A. japonicus* (Fig. 3A). A total of 99 SNPs significantly associated with sex were identified at the threshold of *P* < 1-e6. To provide insights into the potential sex chromosomes, we summarized the chromosome-level statistics of sex-associated SNP density per unit chromosome length (Supplemental Table 4, Fig. 3B). These SNPs were located on multiple chromosomes, which were mainly located on chromosome 4 (24.8%), chromosome 9 (10.7%), chromosome 17 (10.4%), and chromosome 18 (14.1%).

We used the SnpEff program to predict the functional effects of these significant 99 SNPs. These sex-associated SNPs were categorized into four impact groups—high, moderate, low, and modifier. There was one high-impact effect, eleven moderate-impact effects, five low-impact effects, and 129 modifier-impact effects (Supplemental Table 5). The high-impact and moderate-impact effects were all located within the splice site region and exon regions, annotated in six uncharacterized genes (AJ_036483, AJ_017590, AJ_018082, AJ_031977, AJ_033701, AJ_043880). However, the majority of SNPs of the modifier-impact effects are located in intergenic regions. The results about potential functional effects of SNPs were list in Supplemental Table 6.

### Identification of candidate sex-determination region

**Fig. 4:**
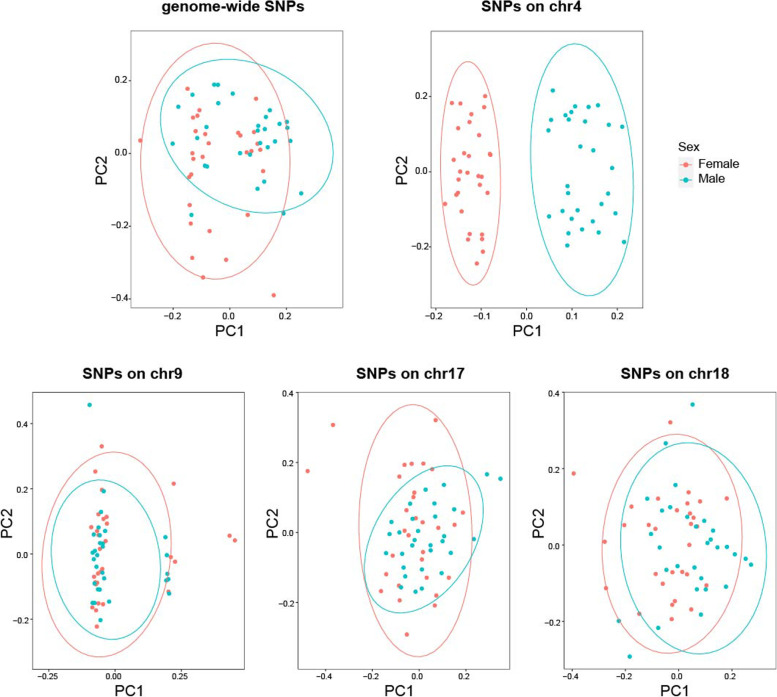
Principal component analysis of 30 females and 30 males using different sets of SNPs

The principal component analysis (PCA) of 60 individuals (30 females and 30 males) with genome-wide SNP set failed to distinguish two sexes (Fig. 4). We then conducted the PCA analysis with chromosome-specific SNPs sets and observed only chromosome 4-specific SNPs clearly distinguishing two sexes, as compared to the additional chromosomes with significant sex-associated locus (chromosome 9, 17, and 18). Using chromosome 4-specific SNPs, 30 females and 30 males were clustered into two groups, suggesting that the genetic variations between females and males were mainly located in chromosome 4.

**Fig. 5:**
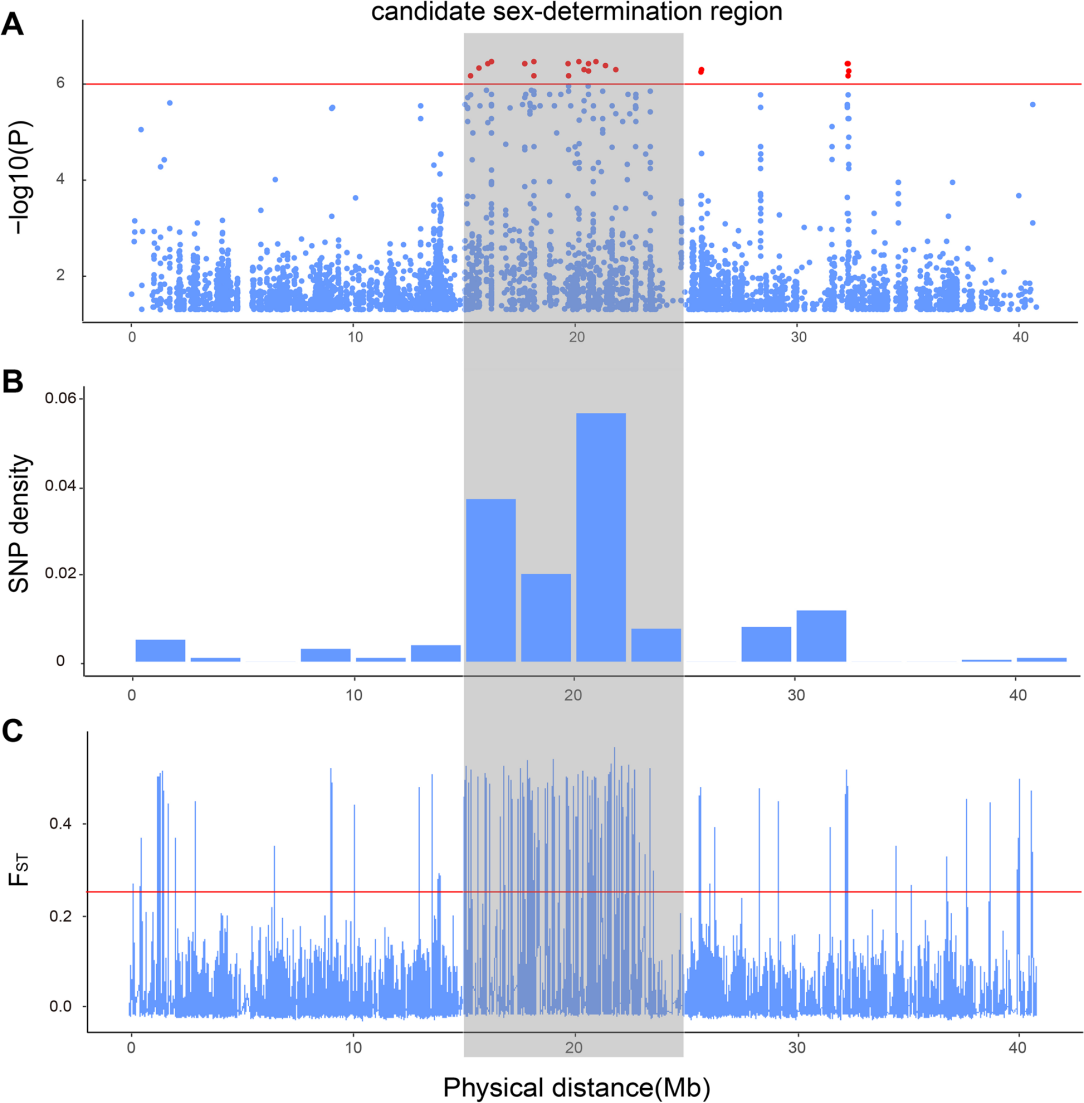
Identification of candidate sex determination regions on chromosome 4. (**A**) GWAS analysis associated with sex of *A. japonicus*. The x-axis depicts the physical location of SNPs, and the y-axis depicts the − log10 (P). The red threshold line represents *P*-value = 1-e6. Red dots represent outlier values of P-value (*P* < 1-e6) (**B**) Distribution of sex-specific SNP density with a sliding window of 2 Mb. (**C**) Distribution of F_ST_ values estimated between females and males. The x-axis depicts the physical location of SNPs, and the y-axis depicts the F_ST_ values. The red threshold line represents F_ST_ = 0.25

In the present study, GWAS studies showed that the physical region on chromosome 4: 15-25 Mb harbored the highest number of sex-associated SNPs (Fig. 5A). Given that, this region might be the candidate SDR in *A. japonicus*. To provide more evidence, we then conducted two methods, one is to isolate the sex-specific SNPs and observe their distribution, and the other is to calculate the F_ST_ values and identify candidate regions with strong selective signatures.

A total of 865 sex-specific SNPs were detected by the custom perl script [55]. Considering the existence of missing data and low coverage depth for certain SNPs, the sex-specific SNPs were required with the overall observed heterozygosity ≥ 0.75 and no heterozygotes in another sex [55]. The genotypes of sex-specific SNPs were listed in Supplemental Table 7. A total of 476 sex-specific SNPs showed heterozygous in more than 75% individuals of tested males and showed homozygous in females. This result indicates that *A. japonicus* might have a XX/XY sex determination system. The genome distribution of sex-specific SNPs was listed in Supplemental Table 8. Approximately 43.12% of sex-specific SNPs were located on chromosome 4, followed by chromosome 10 (10.52%). We then conducted functional annotation of these sex-specific SNPs using SnpEff. There are exon region effects, including missense effects, synonymous effects, and splice region effects. And most SNPs might have potential modifier effects, which were located on the intergenic regions, intron regions, upstream (downstream) regions, and 3' (5') UTR regions (Supplemental Table 9).

Interestingly, most sex-specific SNPs (78.02%) of chromosome 4 are condensed within the physical position from 15 to 25 Mb (Fig. 5B). The candidate region was consistent with our GWAS results. The distribution of sex-specific SNPs on chromosome 4 was in Supplemental Table 10. As shown in Fig. 5C, the above regions showed higher F_ST_ values than adjacent regions. All the evidence indicated the candidate sex determination region was located at 15-25 Mb on chromosome 4 (Fig. 5). We then searched for chromosome 9, 17, and 18, and the sex-associated SNPs identified by GWAS were scattered on the chromosome and only spanned limited regions (Supplemental Fig. 1). Here in this study, the evidence indicated that the sex determination locus might be located on chromosome 4, and the 15–25 Mb region might be a candidate SDR. However, the significant SNPs from the other peaks around 16 Mb and 33 Mb on chromosome 4, and other chromosomes have potential functional effects on genes (e.g. amino acid changes) at different impact levels, which might be also important for sex determination and differentiation of *A. japonicus*.

### Functional annotation of candidate genes for sex determination

**Fig. 6:**
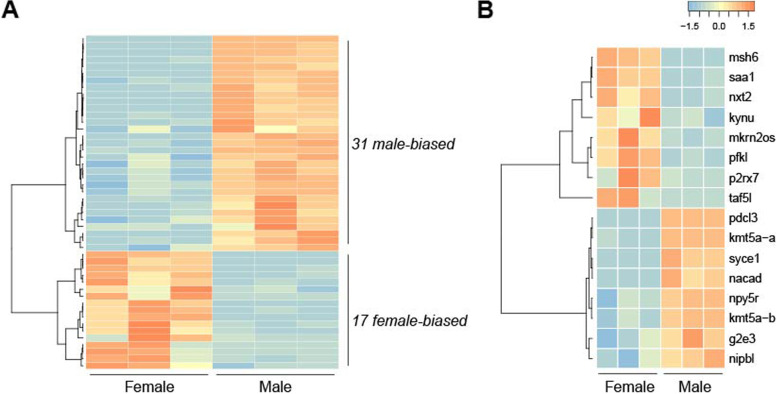
Heatmap of the differentially expressed genes. (**A**) DEGs between female and male gonads within candidate sex determination region. (**B**) The protein-coding genes of DEGs between female and male gonads within candidate sex determination region. Orange represents high expression levels, while blue represents low expression levels

To identify sex-biased genes and candidate sex determination genes within candidate sex determination regions, we analyzed the RNA-Seq dataset of sexually mature female and male gonads (Huang et al., 2021). We identified 13,844 significantly differentially expressed genes (DEGs) with a cutoff of log_2_|FoldChange|> 1 and padj < 0.05, in which 6,890 of them were up-regulated, and 6,954 were down-regulated (Supplemental Table 11). The top enriched KEGG pathways of DEGs were genetic information processing, translation, cellular processes, transport, and catabolism (Supplemental Table 12). In the candidate SDR, we identified 48 DEGs, including 31 male-biased transcripts and 17 female-biased transcripts (Fig. 6A and Supplemental Table 13). Among 16 protein-coding genes, 8 female-biased genes were msh6, saa1, nxt2, kynu, mkrn2os, pfkl, p2rx7, taf5l, and 8 male-biased genes were pdcl3, kmt5a-a, syce1, nacad, npy5r, kmt5a-b, g2e3, nipbl (Fig. 6B and Supplemental Table 14). In the candidate sex determination region, five male-biased genes (kmt5a-a, kmt5a-b, g2e3, nipbl, and syce1) and two female-biased genes (msh6 and taf5l) showed sex-specific expression patterns in other species or associated with gonad function and reproduction. Some other genes within candidate SDR might be differentially expressed and exert essential roles during the early stages that are critical for sex determination and differentiation of *A. japonicus*. The gene annotation within the candidate SDR was listed in Supplemental Table 15.

A Venn diagram was built to investigate shared annotated genes from GWAS studies, sex-specific SNPs analysis, transcriptome analysis of female and male gonads, and genes within candidate SDR (Supplemental Fig. 2 and Supplemental Table 16). As a result, a total of four uncharacterized genes, including AJ_008980, AJ_008729, AJ_008834, and AJ_008676, were shared among these four groups. Two genes associated with sexual function were identified by GWAS study, clca1 (also annotated by sex-specific SNPs) and abca1. Additionally, seven genes were identified using sex-specific SNPs, including g2e3, herc4, mcm8, osr2, vcl, man2a2, and pif1. Among these genes, g2e3 and herc4, were located within the candidate sex determination region. Ten additional genes were identified within the candidate sex determination region, including zbed1, rnf111, cdpk4, recq, ccr1, vps11, nxpe4, mrp5, thap9, and polr3b.

## Discussion

In this study, we assembled a chromosome-level genome and re-sequenced female and male populations to understand the sex determination mechanism of *A. japonicus*. Based on that, we integrated a genome-wide association study and the distribution characteristics of sex-specific SNPs and fixation index F_ST_ to predict the sex-determination locus of *A. japonicus*. Further, we focused on the identification of candidate sex determination genes by combining GWAS studies, sex-specific SNPs analysis, and transcriptome analysis of female and male mature gonads.

In previous studies, the scaffold-level genome assembly of *A. japonicus* has been published and applied to study aestivation, regeneration, saponin biosynthesis, and morphological evolution [39, 60]. However, identifying the sex determination locus and investigating the sex determination mechanism in sea cucumber required chromosome-level genome assembly. Therefore, based on the published scaffold-level genome assembly [39], we constructed a chromosomal-level genome assembly AJH1.0 using Hi-C technology (Fig. 2). The size of the chromosome-level genome assembly AJH1.0 was 767 Mb, approximately 95.28% of the size of the scaffold-level genome assembly of Zhang et al. (2017). Moreover, the chromosome-level genome included a high proportion (90.3%) of BUSCO, suggesting the completeness of the new assembly. This genomic resource will be a powerful resource for genomic, genetic, and epigenetic study in *A. japonicus* and helpful for selective breeding and genetic improvement of *A. japonicus* in aquaculture use.

The chromosome-level genome assembly AJH1.0 consisted of 23 chromosomes with lengths ranging from 22.4 to 60.4 Mb (Fig. 1). Our knowledge of chromosome number in *A. japonicus* has been developed in the last two decades. In 1997, Xu et al. reported that the diploid chromosome number of *A. japonicus* was 40 (2n = 40) [61]. However, in 2008, Okumura identified a new chromosome number of 44 (2n = 44) in *A. japonicus* and explained that the difference in chromosome number is due to intraspecific variation of *A. japonicus* [27]. In 2011, Tan et al. from another research team believed that the chromosome number of *A. japonicus* was 46 (2n = 46) through karyotype analysis of higher sampling numbers (100 individuals) [62]. Tan et al. suggested that the differences in chromosome number are mainly due to the different experimental processing and conditions when preparing slices. The genetic map of *A. japonicus* predicted 22 linkage groups [33]; however, in the present study, the contact map of Hi-C interactions among 23 chromosomes was ideal compared with that among 22 chromosomes. Therefore, regarding previous observation of karyotype analysis in Tan et al. (2011) and ideal Hi-C interactions among chromosomes, our chromosome-level genome included 23 chromosomes.

Recently, genome-wide association study (GWAS) has become increasingly popular in studies on sex determination mechanisms, which is helpful for the identification of sex determination locus, sex-linked markers, and candidate sex-determination genes [63–67]. In *A. japonicus*, GWAS analysis was only used in a recent study identifying genomic locus associated with body color variation [68]. We conducted GWAS associated with sex in sea cucumber and identified sex-associated SNPs in the present study. The most significant 99 SNPs were mainly located on chromosome 4, chromosome 9, chromosome 17, and chromosome 18 (Fig. 3), suggesting that multiple chromosomes are potentially involved in sex determination mechanisms of sea cucumber, and *A. japonicus* might have polygenic sex determination, like two other invertebrates, a mollusc *Pomacea canaliculata* [69] and a copepod, *Tigriopus californicus* [70]. Notably, our PCA results showed that only chromosome 4-specific SNPs clearly distinguish two sexes of sea cucumber, suggesting that the candidate sex determination locus was located on chromosome 4 (Fig. 4).

To narrow down the sex determination locus identified by GWAS, we integrated sex-specific SNPs analysis and F_ST_ measurement. The sex-specific SNPs and F_ST_ values can show the female and male population differences due to genetic variations and structure. In sea cucumber, a physical region of 10 Mb on chromosome 4 may be the candidate sex determination region, which included the highest number of sex-specific SNPs and higher F_ST_ values (Fig. 5). The high density of sex-specific SNPs was a feature of the sex determination locus, which has been observed in a sex determination study of Salangid fish *Protosalanx hyalocranius* [55]. Here, genomic regions with high F_ST_ values tend to coincide with sex determination locus identified by GWAS (Fig. 4). Similar results have been demonstrated in other species, such as *Larimichthys crocea* [71] and *Salix dunnii* [72]. In the candidate SDR, the frequencies of SNPs and INDELs in the AJH1.0 genome assembly showed interesting patterns, which were lower as compared with other genomic regions (Supplemental Fig. 3). However, there are no obvious patterns in chr9, chr17, and chr18. The explanation of such special pattern in the candidate SDR of *A. japonicus* still requires further analysis.

Notably, one sex-specific tag identified in the previous study of *A. japonicus* was at the physical position of 24,970,199 bp-24,970,730 bp on chromosome 4 by local blast [29], which is located in the candidate SDR. In the present study, we conducted GWAS study to identify sex determination locus, but we might exclude some female-specific sequences or variants when analyzing based on a reference genome from a male individual. Due to the absence of haplotype genomes of male and female individuals, it’s a challenge for us to identify female-specific or male-specific region. In future study, the female or male specific region remains to be revealed using other molecular technologies.

After examining the genotypes of sex-specific loci, we suggested that *A. japonicus* has an XX/XY sex determination system. In genome-wide, a total of 476 sex-specific SNPs showed heterozygous in more than 75% of tested males and showed homozygous in females. This is consistent with the results obtained by 2b-RAD sequencing, in which the genotypes of ten SNP markers showed female homogamety and male heterogamety [29]. Likewise, in tropical sea cucumber *Stichopus monotuberculatus*, the sex determination system is also predicted to be XX/XY by comparing the k-mer distribution and mapped rates to the reference genome between females and males [73]. Further analyses in other species of holothuroidea are required to investigate whether the XY system is a shared sex determination system of holothuroidea. A recent study in sea urchins *Mesocentrotus nudus* hypothesized that sea urchin owns a ZZ/ZW sex determination system [65]. Therefore, the sex determination systems seem to be diverse in echinoderms.

Up to date, few studies have been focused on sex-associated genes in *A. japonicus* [74]. To provide insights into the candidate sex-associated gene list, we found a set of genes were associated with sex differentiation or gonadal development. The gene clca1 and abca1 identified by GWAS, have been identified to be involved in individual masculinization in mice [75] and SRY-regulated steroid metabolism in humans [76], respectively. Seven genes identified by sex-specific SNPs were classified to sex differentiation and gonadal function, including g2e3 [77], herc4 [78], mcm8 [79], osr2 [80], vcl [81], man2a2 [82], and pif1 [83]. Notably, g2e3 was located within the candidate sex determination region, which also showed sexually differential expression between female and male gonads. G2e3 was an ancestral gene for a male germline-specific gene phf7 in the fruit fly, which could regulate the male germline sexual fate and spermatogenesis [77]. Herc4 is annotated with sex-specific SNPs and located within candidate sex determination region. Zheng et al. (2008) reported that herc4 was highly expressed in testis, which was involved in reducing the motility of sperm and causing infertility [78]. Therefore, g2e3 and herc4 genes were inferred to be two candidate genes for our future functional research.

To gain knowledge of gene function within the candidate SDR of *A. japonicus*, we analyzed an RNA-Seq dataset of sexually mature female and male gonads [37]. A KEGG enrichment analysis of sexually differentially expressed genes were conducted to identify potential role in sex determination and differentiation. The functions of DEGs within the candidate SDR were noteworthy; they were identified associated with gonadal function and reproduction processes. Five male-biased genes, including g2e3 (as mentioned above), nipbl [84], syce1 [85, 86], kmt5a-a and kmt5a-b [87], and female-biased genes, msh6 [88] and taf5l [89]. Among these genes, two copies of kmt5a (kmt5a-a and kmt5a-b) in male gonads of sea cucumber showed significantly higher expression. This was consistent with a self-fertilizing mangrove rivulus fish, whose kmt5a were observed with notably higher expression levels in male testes as compared with hermaphrodite ovotestes [87]. Multiple genes within the sex determination locus were found involved in gonadal function, possibly suggesting a functional hub for sex determination and differentiation in the sea cucumber.

## Conclusion

With the development of novel sequencing technologies, a more significant number of echinoderm genomes have been sequenced, allowing the exploration of the sex determination mechanism of echinoderms. In the present study, we integrated genome-wide association study, analyses of sex specific SNPs and F_ST_ values, a 10 Mb candidate sex determination region was identified in the sea cucumber. Our results indicated that *A. japonicus* potentially has a XX/XY sex-determination system. Considering the paradoxical diversity of sex determination systems in invertebrates and limited knowledge of sex determination gene (sry gene in mammals, dmrt1 gene in birds) and mechanism in echinoderms, in the future studies, we will use new sequencing techniques to assemble male and female haplotypes separately and investigate transcriptomic profiles during the critical period of sex determination and early gonadal development. This will bring novel insights into gene regulation during primitive gonadogenesis and differentiation and identification of master sex determination gene in sea cucumber. In the sea cucumber industry, investigation of molecular mechanisms will be helpful for artificial fertilization and precise breeding.

## Supplementary Information

**Additional File 1.** 

**Additional File 2.** 

**Additional File 3.** 

**Additional File 4.**

**Additional File 5.** 

**Additional File 6.** 

**Additional File 7.** 

**Additional File 8.**

**Additional File 9.** 

**Additional File 10.**

**Additional File 11.** 

**Additional File 12.** 

**Additional File 13.** 

**Additional File 14.** 

**Additional File 15.**

**Additional File 16.** 

**Additional File 17.**

## Data Availability

The sequencing data that support the findings of this study are openly available in the NCBI Sequence Read Archive (SRA) under BioProject Accession No. PRJNA812362, and the China National GeneBank DataBase (CNGBdb) under BioProject Accession No. CNP0002776 [90].
