# Supplementary material for: Identification of sex determination locus in sea cucumber Apostichopus japonicus using genome-wide association study
Source: BMC Genomics. 2022 May 23;23:391. doi: 10.1186/s12864-022-08632-3 (PMC9128100; doi:10.1186/s12864-022-08632-3)

**Supplemental Figures**

**Supplemental Figure 1.** GWAS analysis associated with sex of *A. japonicus* on chromosome 9, 17, 18. The x-axis depicts the physical location of SNPs, and the y-axis depicts the −log10 (P). The red threshold line represents P-value =1-e6. Red dots represent outlier values of P-value (P<1-e6).


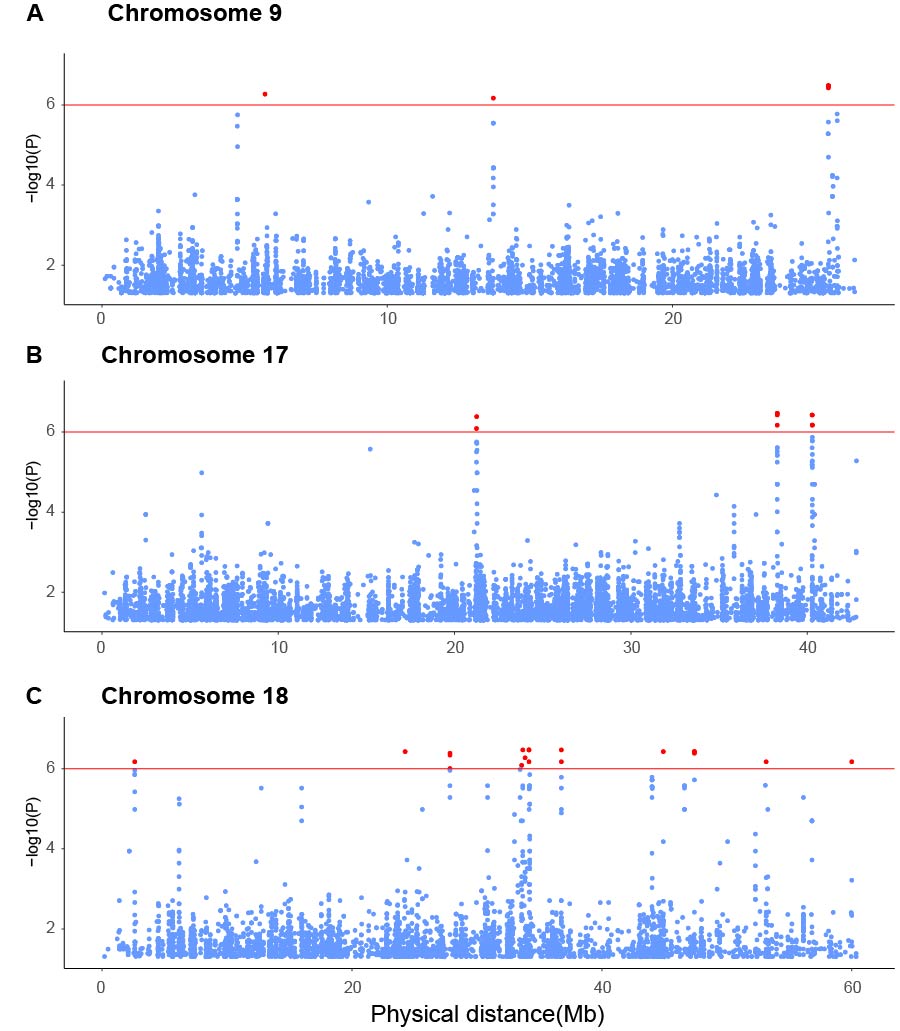


**Supplemental Figure 2.** Venn diagram showed intersection of annotated genes from GWAS studies, sex-specific SNPs analysis, transcriptome analysis and genes within SDR.


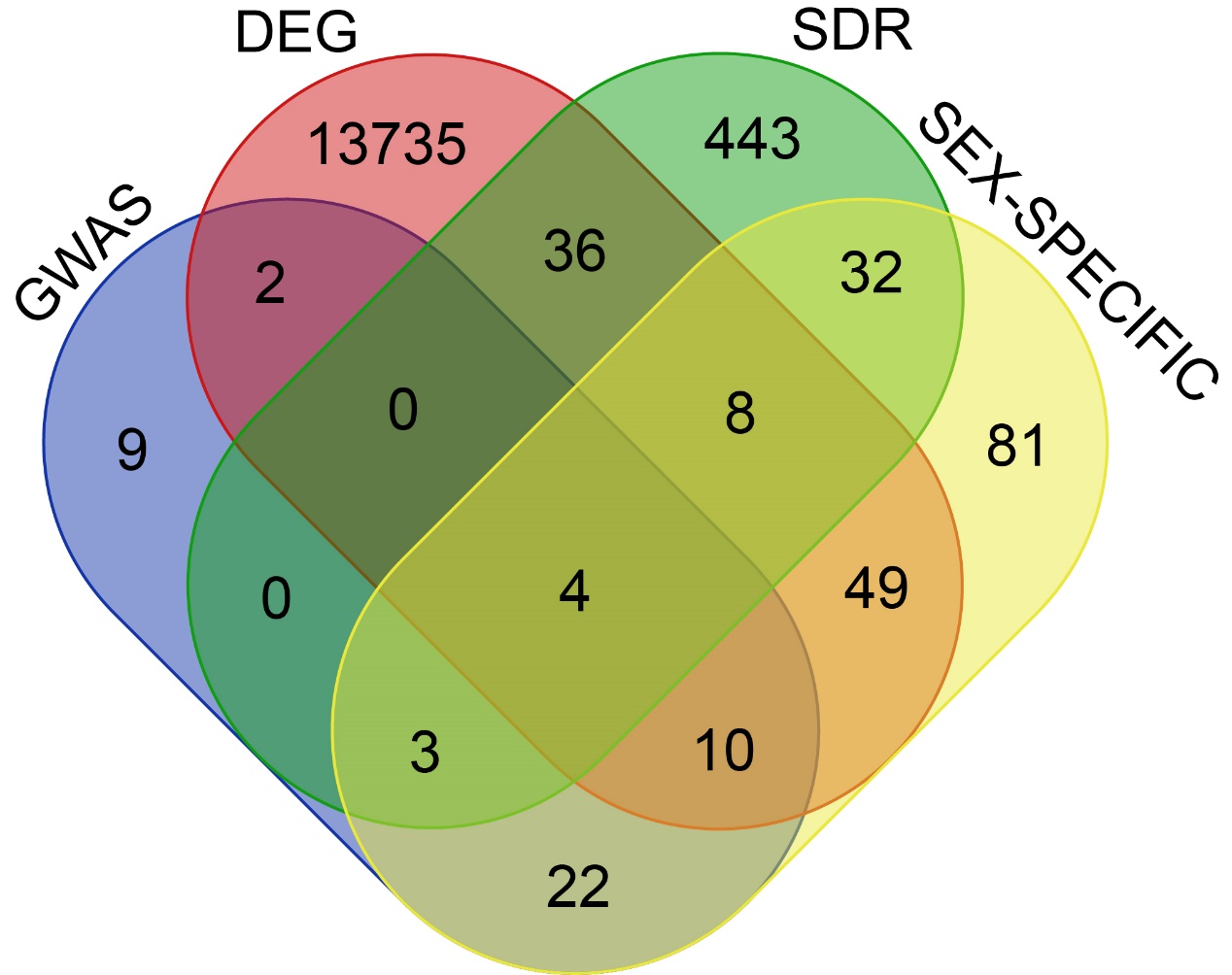


**Supplemental Figure 3.** Circos plot of the global landscape of SNP and INDEL frequency, and sex determination locus in *A. japonicus*. The orange shading indicates important chromosomes contributing to sex determination of *A. japonicus*.

The tracks from outside to inside represent:

(1) 23 chromosomes of AJH1.0 genome assembly (Mb).

(2) Distribution of INDEL density with a sliding window of 500kb.

(3) Distribution of SNP density with a sliding window of 500kb.

(4) Distribution of sex determination locus identified by GWAS.


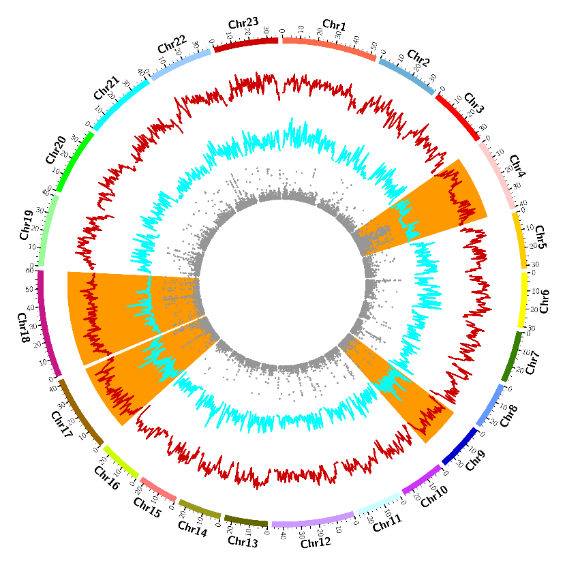

Supplement: Supplementary file 1 — Additional File 1. [file 12864_2022_8632_MOESM1_ESM.docx]
